# Children’s Dental Sealant Use and Caries Prevalence Affected by National Health Insurance Policy Change: Evidence from the Korean National Health and Nutrition Examination Survey (2007–2015)

**DOI:** 10.3390/ijerph16152773

**Published:** 2019-08-03

**Authors:** Minsung Sohn, Sujin Park, Sungwon Lim, Hee-Jung Park

**Affiliations:** 1 Department of Health and Care Administration, The Cyber University of Korea, 106 Bukchon-ro, Jongno-gu, Seoul 03051, Korea; 2 Division of Hospital Management Support, Seoul Health Foundation, 31 Maebongsan-ro, Mapo-gu, Seoul 03909, Korea; 3 School of Nursing, University of Washington, Health Sciences Building, Room T-507, 1959 NE Pacific Street, Seattle, WA 98195, USA; 4 Department of Dental Hygiene, College of Health Science, Kangwon National University, 346 Hwangjo-gil, Dogye-up, Samcheok-si, Gangwon-do 25945, Korea

**Keywords:** dental insurance, dental health services, health policy, pit and fissure sealants

## Abstract

We evaluated the effect of the National Health Insurance (NHI) policy including dental sealant on changes in the prevalence of sealant and caries, and examined how NHI affected sealant utilization and untreated caries in children from diverse income groups in South Korea. We used a multivariate logistic regression analysis to explore the effects of three stages of dental sealant policy (pre-policy: 2007–2009, first post-policy: 2010–2012, and second post-policy: 2013–2015) on the prevalence of dental sealant and untreated caries. Participant data (*N* = 8161, aged 6–14 years) were derived from the Korea National Health and Nutrition Examination Survey (2007–2015). We also conducted subgroup analysis to determine the effects of the NHI policy on dental sealant and untreated caries by income level. Implementation of dental insurance coverage was associated with higher likelihood of using dental sealant (odds ratio (OR) = 1.39 (95% confidence interval (CI): 1.18–1.63) for the first period and OR = 1.58 (95% CI: 1.33–1.87) for the second period) and lower odds of having untreated caries (OR = 0.79 (95% CI: 0.64–0.98) for the first period and OR = 0.65 (95% CI: 0.51–0.83) for the second period) after controlling for covariates. Results revealed that there was a greater prevalence of dental sealant and a lower prevalence of untreated caries in both middle- and low-income households compared to high-income households. The higher prevalence of dental sealant and lower untreated caries after the policy implementation. Moreover, we demonstrated children from low-or middle-income households were more associated with increasing dental sealant use and a declining prevalence of caries.

## 1. Introduction

Dental caries can be preventable, but it is one of the most prevalent chronic diseases among children worldwide [1]. Since dental caries affect most dental and periapical tissues diseases [2], and oral health behaviors in childhood have significant effects on oral health in adulthood particularly [3], it is crucial to control them during children’s development. To solve children’s oral health problem, dental sealant an effective, widespread solution to prevent dental caries [4]. About 90% of cavities in children’s permanent teeth occur on the chewing surfaces of posterior molars, where dental sealants are commonly applied [5]. Additionally, dental sealants are placed in these pits and fissures to fill them, creating a smooth surface that is easy to clean [5]. In practice, it has been reported that sealants prevent about 81% of potential dental caries years after placement [6].

Despite improvement in oral hygiene and the development of medical technology [7], the rate of dental caries in children of South Korea is still ranked higher than many Organization for Economic Co-operation and Development (OECD) countries. The average DMFT (decayed, missing, and filled teeth) index among 12-year-old children in South Korea is 2.08, although children from 21 of the 26 OECD countries had less than 2 DMFT [8]. Accordingly, the Korean government has implemented various efforts to prevent dental caries among children. After the Oral Health Act was passed in 2000, various oral health programs were developed to reduce the incidence of dental caries [7,9]. Since 2002, public oral health programs have implemented community water fluoridation at the community level and provided dental sealants free-of-charge for children from low-income urban and rural areas [10]. Starting in December 2009, health insurance for dental sealant has been available in South Korea, and health insurance coverage have been provided for the first molars of children aged 6–14 years [10]. Subsequently, health insurance coverage benefits have been expanded to include the second molars as well as the first molars, and the benefits extended to children under 6 years of age in October 2012 [10]. In May 2013, the second protection coverage expanded to include children under 18 years of age [10].

In previous studies, public insurance programs have been revealed to be effective in increasing preventive dental visits and reducing costs related to unmet dental needs [11]. For example, in the United States, the Medicaid or the State Children’s Health Insurance Program (SCHIP) system offered preventive services (e.g., oral examinations and restorative services), which aimed to improve dental access for some low-income children and reduce oral health disparities [12]. In the same vein, some studies have analyzed the elimination of dental benefits and the resulting increase in the use of medical settings for dental problems [13]. However, there could be differences in insurance benefits according to social gradients, as socioeconomic status has been identified as a predictor for oral health and the prevalence of dental disease [14]. Previous empirical research has demonstrated that the expansion of coverage has a positive effect on reducing the gap of medical accessibility and health inequity caused by income level [15] and that increased access to dental sealant assists in reducing the oral health disparities between children in low- and high-income families [16]. One study found that that reduction of co-payment to offset financial barriers had a larger effect on impeding access to dental care, especially economically vulnerable population [17].

Recently, there has been a paper on the change between before and after the expansion of health insurance benefits for dental sealants in Korea [18]. However, no study analyzed the effect of health insurance coverage on the prevalence of dental sealant and caries or the effects by income level on children’s oral health. Therefore, we evaluated the effect of National Health Insurance (NHI) policy on changes in the prevalence of sealant and caries and explored how NHI affected sealant utilization and untreated caries across children from diverse income groups in South Korea.

## 2. Methods

### 2.1. Data Source and Study Population

We used data from the 2007–2015 Korea National Health and Nutrition Examination Survey (KNHANES). The KNHANES is a national representative survey that assessed the health and nutritional status of South Koreans and is a continuous, annual survey program conducted by the Korea Centers for Disease Control and Prevention since 1998 [19]. Each survey year includes a new sample of about 10,000 individuals aged one year and over [19]. The KNHANES used a complex, multistage probability sampling design based on sex, age, and geographic area, using household registries, and was conducted on a triennial basis [20]. This data was obtained through physical examinations, clinical and laboratory samples, personal or family interviews, chronic disease, and related measurement procedures for the development and evaluation of health policies and programs in South Korea [19].

Data from 73,353 people were collected during the KNHANES (2007–2015). In this study, 9091 participants (aged 6–14 years) were included. Of these, 547 people who had missing questionnaire data and 383 who had incomplete for survey weight data were excluded from analyses. Therefore, participants were 8161 children (the inclusion rate was 89.77%). An institutional review board approved this study, which qualified for exempt status due to the analysis of de-identified secondary data (exemption number = KWNUIRB-2018-03-006).

### 2.2. Measurement

Our two primary outcomes were the prevalence of dental sealant use and untreated dental caries. For the prevalence of dental sealant use, we assessed the occurrence of sealed teeth only on fully erupted and healthy (non-carious) molars. Sealant was defined as the existence of pit and fissure sealant in the occlusal surfaces of the permanent first and second molars (yes or no) [7]. The prevalence of untreated dental caries included the present, permanent decayed first and second molars (yes or no). Decayed teeth were defined as having lesions in a pit or fissure, or on a smooth surface, and undermined enamel. Dental caries were based on the diagnostic criteria developed by the World Health Organization’s criteria [21].

Policy change was measured as the key independent variable. Specifically, policy implementation was divided into three time periods: pre-policy (2007–2009), the first period of policy implementation (2010–2012), and the second period of policy implementation (2013–2015).

Covariates were selected based on those identified as being commonly associated with outcomes in previous empirical research [14,22]. We included sex, age, family structure, the region of residence, geographical accessibility to dental professionals, type of health insurance, and income level as covariates.

The covariate of Geographical Accessibility to dental professionals was determined based on the number of dentists per 1000 population in each of the 16 administrative districts (i.e., Seoul, Busan, Daegu, Incheon, Gwangju, Daejeon, Ulsan, Gyeonggi, Gangwon, Chungbuk, Chungnam, Jeonbuk, Jeonnam, Gyeonbuk, Gyeongnam, and Jeju) from 2007 through 2015. This variable was obtained from the e-Provincial Indicators and National Health Insurance Statistical Yearbook, which were collected by Statistics Korea and Health Insurance Review and Assessment Service, of the National Health Insurance service.

Income was defined as the monthly household equivalent income according to the equalized gross annual household income (equivalent household income = monthly household income/square root of the number of household members, after considering sex and age stratum). Household income was categorized into quartiles: low, lower-middle, upper-middle, and upper.

For our analyses, the lower-middle and upper-middle groups were combined to form a single level; therefore, three levels of household income (high, middle, and low) were used. Additionally, dental health behaviors such as self-rated dental health status, dental checkup within a year, and frequency of daily teeth brushing per day were also assessed. A self-report measure of oral health (excellent, good, fair, poor, or very poor) was included in the analysis. However, a 3-level rating was used with ratings of poor or very poor being categorized as “poor”, a rating of fair as “fair”, and ratings of good or excellent as “excellent”. The occurrence of a dental checkup within a year was determined based on the question, “Did you have a regular dental checkup within 1 year prior to the interview?” with “yes” and “no” being the only responses to this question. It was assumed that children who answered yes were regularly visiting a dental office to maintain their oral health despite the absence of apparent dental problems. The frequency of daily teeth brushing per day was divided into “one time and or not at all” and “two times or more”.

### 2.3. Statistical Analyses

The KNHANES utilized a complex and multi-stage probability sample survey that contained primary sampling units (PSU), strata (Kstrata), and sample weights [19]. The PSU were selected from a sampling frame of all census blocks or resident registration addresses [19]. Our analyses incorporated sampling weights to obtain nationally representative estimations and to consider the annual weight for 2007–2015. The differences in prevalence of dental sealant and untreated dental caries per the three stages of dental sealant policy and covariates were examined using chi-square tests. Multivariate logistic regression models were applied to determine the change in the prevalence of dental sealant use and the non-treatment of caries because of policy implementation adjusting for covariates by calculating odds ratios (ORs) with 95% confidence intervals (CIs). A subgroup analysis was conducted to determine how policy affected participants by their income level. All analyses were conducted using SPSS 20.0 (SPSS Inc., Chicago, IL, USA).

## 3. Results

### 3.1. Sample Characteristics

Table 1 shows changes in the covariates over time (i.e., pre-policy, the first period of policy implementation, and the second period of policy implementation). In the second period of policy implementation, the proportion of individuals receiving Medical Aid and reporting excellent self-rated dental health status were lower than pre-policy and first period of policy implementation, while the proportion of the frequency of teeth brushing per day, and the number of dentists per 1000 residents were higher than pre-policy, and first period of policy implementation.

Table 2 provides information on the changes in the outcome variables over time by covariate status. In the dental sealant group, the proportion of prevalence of dental sealant was likely to increase after NHI policy implementation (first period: 37.59% and second period: 33.88%, respectively). Children living with both parents (82.66%) were more likely to have received dental sealant than those living without both parents (17.34%). Children in urban areas (84.00%) and under NHI coverage (95.94%) were more likely to receive dental sealant. With regards to untreated caries, the prevalence of children with treated caries was similar to the prevalence of dental sealant, which increased after the NHI policy implementation (first period: 36.41% and 30.79%, respectively). Children aged 10–14 years (56.63%) were treated more frequently than were children aged 6–9 years (43.37%). Children living with both parents (81.15%) were more likely to be treated for dental caries than those living without both parents (18.85%). Children in urban areas (83.27%) and under NHI coverage (95.73%) were more likely to be treated for dental caries.

**Figure 1:**
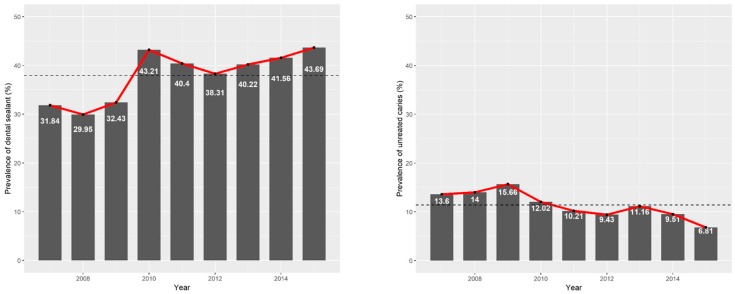
Changes in the prevalence of dental sealant use and untreated caries over time.

Figure 1 shows changes in the prevalence of dental sealant use and untreated caries from 2007 to 2015. The prevalence of dental sealant use was increasing trend, whereas the prevalence of untreated caries was decreasing trend.

### 3.2. Association between Dental Insurance Coverage and Dental Sealant or Untreated Caries

In model I (crude model), the implementation of dental insurance coverage was strongly associated with higher odds of dental sealant use (OR = 1.36 (95% CI: 1.16–1.59) for the first period and OR = 1.60 (95% CI: 1.35–1.89) for the second period) and lower odds of having untreated caries (OR = 0.82 (95% CI: 0.67–0.99) for the first period and OR = 0.61 (95% CI: 0.49–0.76) for the second period) compared with those of pre-policy (Table 3).

In model II (adjusted model), the implementation of dental insurance coverage was strongly associated with higher odds of dental sealant use (OR = 1.39 (95% CI: 1.18–1.63) for the first period and OR = 1.58 (95% CI: 1.33–1.87) for the second period) and lower odds of having untreated caries (OR = 0.79 (95% CI: 0.64–0.98) for the first period and OR = 0.65 (95% CI: 0.51–0.83) for the second period) compared with those of pre-policy after controlling for covariates (Table 3). Children aged 6–9 years had a significantly lower prevalence of using dental sealant (OR = 0.67 (95% CI: 0.60–0.74)) and having untreated caries (OR = 0.28 (95% CI: 0.23–0.34)) than those aged 10–14 years. Children living without (vs. with) both parents had significantly lower odds of having used dental sealant (OR = 0.76 (95% CI: 0.65–0.89). In contrast, children living without both parents (vs. with) had higher odds of having untreated caries (OR = 1.53 (95% CI: 1.22–1.90)). Poor or fair self-rated dental health status was associated with higher odds of dental sealant (OR = 1.48 (95% CI: 1.23–1.77) for poor and OR = 1.35 (95% CI: 1.16–1.57) for fair) and lower odds of untreated caries (OR = 0.23 (95% CI: 0.18–0.30) for poor and OR = 0.37 (95% CI: 0.31–0.44) for fair). In addition, children who had not received a dental checkup (compared to those who had) within the past year had lower odds of having received dental sealant and greater odds of having untreated caries (OR = 0.70 (95% CI: 0.62–0.79) and OR = 1.35 (95% CI: 1.14–1.60), respectively).

### 3.3. Subgroup Analysis per Household Income

We revealed a difference by household income in the prevalence of dental sealant use, showing a significantly greater prevalence of having been treated with dental sealant in the first (OR = 1.29 (95% CI: 1.04–1.60) for middle-income and OR = 1.83 (95% CI: 1.35–2.47) for low-income) and second period (OR = 1.58 (95% CI: 1.26–1.99) for middle-income and OR = 2.05 (95% CI: 1.50–2.81) for low-income) for middle- and low-household income groups. In addition, the results also reflected a household income difference in the prevalence of having untreated caries, showing a lower prevalence of untreated caries in the first (OR = 0.67 (95% CI: 0.49–0.90) for middle-income) and second period (OR = 0.62 (95% CI: 0.44–0.86) for middle-income and OR = 0.58 (95% CI: 0.38–0.86) for low-income) for middle- and low-income households (Table 4).

## 4. Discussion

This study was a first attempt to examine the relationship between the implementation of health insurance coverage under the NHI, including the use of dental sealant and the prevalence of caries, using KNHANES data from 2007–2015. Additionally, we analyzed the extent that the policy affects children depending on their household income.

We verified that the prevalence of dental sealant use increased from the pre-policy period to the first and second period of policy implementation (2010–2012 and 2013–2015, respectively), after adjusting for potential covariates (including household income). A recent study conducted in the US reported similar findings and noted the importance of accessible dental sealant utilization through Medicaid, with the rate of visits for sealant treatment increasing significantly from 3% to 11% after the introduction of the policy to permit non-dentists to become certified Medicaid providers in public health settings [23]. The need to minimize barriers to dental care access has led to the continued investigations to identify innovative programs and policies that could improve sealant utilization in high-risk children. Further, public insurance programs such as Medicaid and SCHIP in the US have been effective in increasing dental visits and in decreasing the costs associated with unmet dental needs [11]. In addition, State Medicaid/SCHIP policy changes aimed at promoting greater preventive care receipt may be associated with more prevalent annual preventive dental visit among publicly insured children [24].

More importantly, we showed that the number of children with untreated dental caries significantly decreased after the policy change. Our findings may also be congruent with evidence showing that system-level factors, such as the health delivery system, influence childhood caries at the population level [25]. Moreover, these results were consistent with the previous findings of Decker and Lipton [26] as well as Sommers and colleagues [27] who contended that providing public dental insurance would contribute to the reduction in existing oral health problems because of improvements in regular dental exams and cleanings. These indicated that the decline in the incidence of untreated caries might be related to the inexpensive and easy access to preventive dental services after the coverage of dental sealant utilization under the NHI began. It is, therefore, possible that the increasing sealant utilization may lead to the forgoing of dental services for developing caries. However, some studies [7,22] have reported that the occurrence of childhood dental caries might have decreased owing to other social determinants associated with the prevalence of dental caries (e.g., infant mortality rate, dental health education, rapid industrialization, and urbanization) and behaviors (e.g., visit to the dentist, exposure to fluoride, specific types of nutrients and food intake) rather than from dental sealant utilization in South Korea.

Another key finding was that children in low- and middle-income households were more likely to have sealed teeth as compared to those from high-income households during 2007–2009. Moreover, children in low- and middle-income households were less likely to have untreated caries as compared to those from high-income households. Notwithstanding these findings, it is critical to note that public dental insurance system is considered to be the most feasible approach to eliminating income disparities in the use of dental care services [11,15,17]. Zhou et al. [11] has confirmed that public dental insurance would contribute to resolving the income disparity in dental care access for the poor vulnerable and deprived population. Based on current evidence, this implies that public health interventions at the national level play a key role in increasing the accessibility of dental care services and decreasing dental caries among socially marginalized individuals.

The effectiveness of the public dental health insurance scheme as a means of warding off the high out-of-pocket costs of seeking dental care in the vulnerable population has been disputed in previous studies. A recent study [28] has reported that insurance coverage expansion that included a preventive scaling policy in South Korea was paradoxically more effective for adults with high socioeconomic status, as defined by income and education level. The present study noted that the effect of the policy was greater among individuals with higher levels of income or education, in contrast with the policy objective of benefiting all participants in the NHI system. These results were inconsistent with our findings. The main reasons behind these results were that sealant and periodontal scaling in dental policies inherently differ from the dental care benefits under NHI system.

Although it is difficult to present straightforward evidence that the effects of the policy were seen only in the low- or middle-income groups, we considered several possibilities. One plausible explanation is that high-income groups were likely to have already received dental sealant or caries treatment regardless of the policy reform. Therefore, access to sealant utilization as well as the caries prevalence among the high-income group, may not have changed significantly after the introduction of the policy. Future studies should attempt to uncover a consistent explanation for children’s dental care access and dental health treatment among high-income households affected by the policy change.

This study had several limitations. First, our results were derived by employing a cross-sectional design; therefore, it is difficult to clarify the causality of the policy change and its outcomes. Previous research has suggested that a quasi-experimental design, such as difference-in-difference methodology or an interrupted time-series analysis, may help solve the endogeneity problem by comparing time trends among target and control groups [29], thus highlighting the need for research using such designs. Second, we were unable to include other potential covariates (e.g., parents’ socioeconomic factors and oral health knowledge) due to missing paternal information. In particular, given previous findings that higher educated (vs. less) patients were more likely to receive dental care [30], it is assumed that parents’ educational level might mediate or moderate the effect of the policy change-outcomes. Therefore, it is necessary to consider parents’ socioeconomic status. Lastly, further study should analyze the moderating roles of the interaction between oral health policy and income level in models to better understand the moderating effects of the policy in the process of income impacting dental use and health status.

Despite these limitations, we provided some novel insights on ways to assess the impact of policy implementation. A key finding from these analyses was that the intent of the policy—to improve dental sealant utilization under the new insurance system—appears to have had a substantial positive effect. In addition, the overall use of dental sealants increased, and the incidence of untreated caries decreased in low- and middle-income households post-policy change. Therefore, policymakers should continue to explore strategies directly aimed at children in South Korea to improve their use of and access to public dental services, thus promoting their dental health.

## 5. Conclusions

Our results highlighted the potential consequences of reform enacted upon public healthcare systems, including the promotion of dental sealant use and boosting children’s access to dental care, improving their dental health in South Korea. The higher prevalence of dental sealant utilization and lower incidence of untreated caries in the wake of this policy implementation implies the importance of such a large commitment required of the NHI system. Moreover, we demonstrated that children from low- or middle-income households were more associated with increasing dental sealant use and a declining prevalence of caries. The shift towards better coverage through public insurance is necessary for the facilitation of the use of pediatric dental care services, as well as to mitigate the caries epidemic in vulnerable populations.

## Figures and Tables

**Table 1:** Changes in the covariates over the three time periods.

Category	Pre-Policy (2007–2009)		First Period of Policy Implementation (2010–2012)		Second Period of Policy Implementation (2013–2015)	
	Unweighted Frequency (Weighted %)		Unweighted Frequency (Weighted %)		Unweighted Frequency (Weighted %)	
Sex						
Girl	1544	(47.5)	1339	(48.0)	995	(49.0)
Boy	1701	(52.5)	1490	(52.0)	1092	(51.0)
Age (years)						
6–9	1466	(40.5)	1222	(38.6)	950	(41.2)
10–14	1779	(59.5)	1607	(61.4)	1137	(58.8)
Family structure						
Living without both parents	483	(15.7)	461	(19.1)	560	(26.4)
Living with both parents	2762	(84.3)	2368	(80.9)	1527	(73.6)
Region of residence						
Rural	586	(15.6)	398	(17.4)	381	(17.6)
Urban	2659	(84.4)	2431	(82.6)	1706	(82.4)
Type of health insurance						
Medical aid	176	(5.3)	112	(5.3)	62	(3.3)
NHI	3069	(94.7)	2717	(94.7)	2025	(96.7)
Household income						
Low	801	(24.9)	674	(29.1)	482	(23.1)
Middle	1603	(49.1)	1432	(49.9)	1066	(51.9)
High	841	(26.0)	723	(21.0)	539	(25.0)
Self-rated dental health status						
Poor	680	(20.1)	656	(22.4)	593	(28.4)
Fair	1797	(55.8)	1670	(57.3)	1186	(56.8)
Excellent	768	(24.1)	503	(20.3)	308	(14.8)
Dental checkup within a year						
No	1247	(38.1)	1148	(42.8)	715	(34.2)
Yes	1998	(61.9)	1681	(57.2)	1372	(65.8)
Frequency of teeth brushing per day						
0–1	444	(13.9)	270	(10.7)	163	(7.8)
Above 2 times	2801	(86.1)	2559	(89.3)	1924	(92.2)
Number of dentists per 1000 residents (Mean/SD)	0.392	(0.004)	0.416	(0.004)	0.432	(0.005)

**Table 2:** Changes in the outcome variables over time by covariates.

Category	Prevalence of Dental Sealant Use					Prevalence of Untreated Caries				
	No		Yes		*p*-Value	No		Yes		*p*-Value
	Unweighted Frequency (Weighted %)		Unweighted Frequency (Weighted %)			Unweighted Frequency (Weighted %)		Unweighted Frequency (Weighted %)		
NHI-insurance coverage: dental sealant										
Second Period (2013–2015)	1218	(27.39)	869	(33.88)	<0.001	1891	(30.79)	196	(23.03)	<0.001
First Period (2010–2012)	1673	(35.75)	1156	(37.59)		2527	(36.41)	302	(36.59)	
Pre-policy (2007–2009)	2229	(36.86)	1016	(28.53)		2771	(32.80)	474	(40.38)	
Sex										
Girl	2427	(48.25)	1451	(47.95)	0.824	3404	(48.00)	474	(49.07)	0.587
Boy	2693	(51.75)	1590	(52.05)		3785	(52.00)	498	(50.93)	
Age (years)										
6–9	2453	(42.99)	1185	(35.02)	<0.001	3439	(43.37)	199	(16.79)	<0.001
10–14	2667	(57.01)	1856	(64.98)		3750	(56.63)	773	(83.21)	
Family structure										
Living without both parents	1019	(21.78)	485	(17.34)	<0.001	1248	(18.85)	256	(28.95)	<0.001
Living with both parents	4101	(78.22)	2556	(82.66)		5941	(81.15)	716	(71.05)	
Region of residence										
Rural	893	(17.40)	472	(16.00)	0.304	1175	(16.73)	190	(17.93)	0.457
Urban	4227	(82.60)	2569	(84.00)		6014	(83.27)	782	(82.07)	
Type of health insurance										
Medical aid	247	(5.10)	103	(4.06)	<0.001	273	(4.27)	77	(7.75)	<0.001
NHI	4873	(94.90)	2938	(95.94)		6916	(95.73)	895	(92.25)	
Household income										
Low	1388	(27.61)	619	(22.95)	<0.001	1618	(24.44)	339	(35.74)	<0.001
Middle	2,570	(50.30)	1531	(50.11)		3679	(51.42)	422	(42.04)	
High	1212	(22.09)	891	(26.94)		1892	(24.14)	211	(22.22)	
Self-rated dental health status										
Poor	1167	(22.06)	762	(25.24)	<0.001	1809	(24.99)	120	(11.14)	<0.001
Fair	2863	(55.67)	1790	(58.48)		4177	(57.89)	479	(48.62)	
Excellent	1090	(22.27)	489	(16.28)		1203	(17.12)	376	(40.24)	
Dental checkup within a year										
No	2104	(41.74)	1006	(33.48)	<0.001	2640	(37.25)	470	(48.41)	<0.001
Yes	3016	(58.26)	2035	(66.52)		4549	(62.75)	502	(51.59)	
Frequency of teeth brushing per day										
0–1	605	(12.24)	272	(8.68)	<0.001	735	(10.47)	142	(13.95)	0.005
Above 2 times	4515	(87.76)	2769	(91.32)		6454	(89.53)	830	(86.05)	
Number of dentists per 1000 residents (Mean/SD)	0.409	(0.003)	0.413	0.004	<0.001	0.411	(0.003)	0.408	(0.005)	<0.001

**Table 3:** Association between dental insurance coverage and using dental sealant and having untreated caries.

Category	Prevalence of Dental Sealant Use				Prevalence of Untreated Caries			
	Model I		Model II		Model I		Model II	
	OR	95% CI	OR	95% CI	OR	95% CI	OR	95% CI
NHI-insurance coverage: dental sealant								
Second Period (2013–2015)	1.60	1.35, 1.89	1.58	1.33, 1.87	0.61	0.49, 0.76	0.65	0.51, 0.83
First Period (2010–2012)	1.36	1.16, 1.59	1.39	1.18, 1.63	0.82	0.67, 0.99	0.79	0.64, 0.98
Pre-policy (2007–2009)	1.00		1.00		1.00		1.00	
Sex								
Girl			0.98	0.87, 1.09			1.07	0.91, 1.26
Boy			1.00				1.00	
Age (years)								
6–9			0.67	0.60, 0.74			0.28	0.23, 0.34
10–14			1.00				1.00	
Family structure								
Living without both parents			0.76	0.65, 0.89			1.53	1.22, 1.90
Living with both parents			1.00				1.00	
Region of residence								
Rural			0.92	0.75, 1.12			1.06	0.83, 1.35
Urban			1.00				1.00	
Type of health insurance								
Medical aid			1.03	0.75, 1.41			1.05	0.70, 1.56
NHI			1.00				1.00	
Household income								
Low			0.78	0.65, 0.94			1.23	0.95, 1.59
Middle			0.82	0.71, 0.94			0.84	0.67, 1.05
High			1.00				1.00	
Self-rated dental health status								
Poor			1.48	1.23, 1.77			0.23	0.18, 0.30
Fair			1.35	1.16, 1.57			0.37	0.31, 0.44
Excellent			1.00				1.00	
Dental checkup within a year								
No			0.70	0.62, 0.79			1.35	1.14, 1.60
Yes			1.00				1.00	
Frequency of teeth brushing per day								
0–1			0.77	0.64, 0.92			1.02	0.79, 1.31
Above 2 times			1.00				1.00	
Number of dentists per 1000 residents (Mean/SD)			0.97	0.76, 1.23			1.03	0.74, 1.45

Note. OR = odds ratio; 95% CI = 95% confidence interval; Ref. = reference group.

**Table 4:** (**a**) Odds Ratio for the prevalence of dental sealant use by household income; (**b**) Odds Ratio for prevalence of having untreated caries by household income.

(**a**)						
**Category**	**Prevalence of Dental Sealant Use**					
	**High**		**Middle**		**Low**	
	**OR**	**95% CI**	**OR**	**95% CI**	**OR**	**95% CI**
NHI-insurance coverage: dental sealant						
Second Period (2013–2015)	1.27	0.94, 1.73	1.58	1.26, 1.99	2.05	1.50, 2.81
First Period (2010–2012)	1.26	0.94, 1.67	1.29	1.04, 1.60	1.83	1.35, 2.47
Pre-policy (2007–2009)	1.00		1.00		1.00	
(**b**)						
**Category**	**Prevalence of Untreated Caries**					
	**High**		**Middle**		**Low**	
	**OR**	**95% CI**	**OR**	**95% CI**	**OR**	**95% CI**
NHI-insurance coverage: dental sealant						
Second Period (2013–2015)	0.78	0.48, 1.28	0.62	0.44, 0.86	0.58	0.38, 0.86
First Period (2010–2012)	0.76	0.49, 1.19	0.67	0.49, 0.90	0.94	0.66, 1.35
Pre-policy (2007–2009)	1.00		1.00		1.00	

Note. OR = odds ratio; 95% CI = 95% confidence interval; Ref. = reference group.
